# Guidelines for the management of myocardial infarction/injury with non-obstructive coronary arteries (MINOCA): a position paper from the Dutch ACS working group

**DOI:** 10.1007/s12471-019-01344-6

**Published:** 2019-11-22

**Authors:** T. F. S. Pustjens, Y. Appelman, P. Damman, J. M. ten Berg, J. W. Jukema, R. J. de Winter, W. R. P. Agema, M. L. J. van der Wielen, F. Arslan, S. Rasoul, A. W. J. van ’t Hof

**Affiliations:** 1 Department of Cardiology, Zuyderland Medical Centre, Heerlen, The Netherlands; 2 Department of Cardiology, location VU University Medical Centre, Amsterdam UMC, Amsterdam, The Netherlands; 3 Department of Cardiology, Radboud University Medical Centre, Nijmegen, The Netherlands; 4 Department of Cardiology, St Antonius Hospital, Nieuwegein, The Netherlands; 5 Department of Cardiology, Leiden University Medical Centre, Leiden, The Netherlands; 6 Department of Cardiology, location Academic Medical Centre, Amsterdam UMC, Amsterdam, The Netherlands; 7 Department of Cardiology, Jeroen Bosch Hospital, ’s-Hertogenbosch, The Netherlands; 8 Department of Cardiology, location Bethesda, Treant Zorggroep, Hoogeveen, The Netherlands; 9 Department of Cardiology, Maastricht University Medical Centre, Maastricht, The Netherlands; 10 Department of Cardiology, Isala Hospital, Zwolle, The Netherlands

**Keywords:** Myocardial infarction, Non-obstructive coronary arteries, MINOCA

## Abstract

Patients with myocardial infarction and non-obstructive coronary arteries (MINOCA), defined as angiographic stenosis <50%, represent a conundrum given the many potential underlying aetiologies. Possible causes of MINOCA can be subdivided into coronary, myocardial and non-cardiac disorders. MINOCA is found in up to 14% of patients presenting with an acute coronary syndrome. Clinical outcomes including mortality, and functional and psychosocial status, are comparable to those of patients with myocardial infarction and obstructive coronary arteries. However, many uncertainties remain regarding the definition, clinical features and management of these patients. This position paper of the Dutch ACS working group of the Netherlands Society of Cardiology aims to stress the importance of considering MINOCA as a dynamic working diagnosis and to guide the clinician in the management of patients with MINOCA by proposing a clinical diagnostic algorithm.

## Introduction

Up to 14% of the patients with acute myocardial infarction (AMI) are found to have non-obstructive coronary arteries, defined as coronary stenosis <50% [1]. The term myocardial infarction (MI) with non-obstructive coronary arteries (MINOCA) has been coined for this clinical entity, which represents a diagnostic and therapeutic dilemma since many patients are discharged without a clear aetiology for the clinical presentation [2].

Despite the fact that this syndrome has been examined in greater depth over the past few years, many uncertainties remain regarding the pathophysiology of the myocardial damage, the clinical features, management and prognosis of these patients. As a result, the patients may be treated inappropriately or not treated at all.

On behalf of the Dutch ACS working group, we discuss the importance of MINOCA and will present a diagnostic algorithm to guide the general and interventional cardiologist which may lead to optimal treatment of this patient cohort.

## Definition

The diagnosis MINOCA requires (1) the presence of an AMI (according to the Fourth Universal Definition of AMI, see Tab. 1), (2) non-obstructive coronary arteries on invasive coronary angiography, defined as no coronary stenosis ≥50% in any potential infarct-related artery, and (3) no clinically overt specific cause for the acute presentation [2, 3].

**Table 1:** Fourth universal definition of myocardial infarction

The fourth universal definition of acute myocardial infarction (AMI) defines AMI as the presence of:
1. acute myocardial injury with clinical evidence of acute myocardial ischaemia, and
2. with detection of a rise and/or fall of cardiac troponin with at least one value above the 99th percentile upper reference limit, and
3. with at least one of the following: – symptoms of myocardial ischaemia – new ischaemic ECG changes – development of pathological Q waves – imaging evidence of new loss of viable myocardium or regional wall motion abnormality in a pattern consistent with an ischaemic aetiology – the identification of a coronary thrombus by angiography or autopsy

Many terms have been coined to describe patients with AMI or acute coronary syndrome (ACS) with normal or near-normal coronary arteries, such as MINOCA, MINCA (MI with normal coronary arteries) [4] and INOCA (ischaemia and no obstructive coronary artery disease) [5].

The term MINOCA is incorporated into the recently published Fourth Universal Definition of AMI. According to this, MINOCA (i.e. *myocardial infarction*) indicates that an ischaemic mechanism is the underlying cause for the myocyte injury. Others have used the term MINOCA in an all-encompassing context to include all patients fulfilling the universal criteria for AMI without obstructive coronary artery disease (CAD) [2].

In light of this, Pasupathy et al. proposed a new term called troponin-positive non-obstructive coronary arteries (TP-NOCA) that includes patients with coronary disorders resulting in ischaemic necrosis, and myocardial and non-cardiac disorders resulting in myocardial injury [6]. Another proposed term is ACSNNOCA (ACS with normal or near-normal coronary arteries) which encompasses all ACS patients with non-obstructive coronary arteries (i.e. MINOCA/MINCA/INOCA).

There is much overlap between all these terms, including type 2 MI [3]. The last-mentioned is also a heterogeneous category that includes pathophysiological mechanisms comparable to MINOCA. From a clinical point of view, it is extremely challenging (directly after coronary angiography) to make a clear distinction between whether the patient with a suspected AMI and non-obstructive coronary arteries suffers from myocardial injury, ischaemia or infarction.

Therefore, as the Dutch ACS working group, we propose that MINOCA should not be considered as a ‘true’ diagnosis, but rather as a clinical dynamic working diagnosis that needs further evaluation. If coronary angiography during a suspected AMI shows non-obstructive coronary arteries and there is no overt cause for the clinical presentation, the working diagnosis MINOCA could be made. In the further evaluation of the underlying mechanism of AMI it is imperative to exclude other clinically overt causes for the elevated troponin (e.g. sepsis, hypotension and pulmonary embolism) and non-ischaemic mechanisms of myocyte injury (e.g. myocarditis). Viewed in this way, which reflects clinical practice best, MINOCA can represent both *myocardial infarction* and *myocardial injury* with non-obstructive coronary arteries.

In this paper, the all-encompassing term MINOCA is used to describe the coronary, myocardial and non-cardiac aetiologies, similar to the position paper on MINOCA of the European Heart Journal [2]. Most importantly, MINOCA should be considered as a dynamic working diagnosis, which should encourage the clinician to further evaluate the underlying mechanism(s) in order to achieve patient-specific treatments.

## Clinical characteristics and assessment of MINOCA

MINOCA can present with ST-elevation MI (STEMI) (approximately 1/3) or non-STEMI (approximately 2/3) [1]. As stated before, the causes of MINOCA can be subdivided into coronary, myocardial or non-cardiac related disorders (Tab. 2). In the 1980s, DeWood et al. reported that approximately 10% of patients with MI were found to have non-obstructive CAD [7]. Currently, the prevalence may be even higher in the era of high-sensitivity cardiac troponin assays, because of their lower specificity to diagnose acute MI. A systematic review by Pasupathy et al. indicates a MINOCA prevalence of 6% in ACS patients, with a wide range of 1–15% [1, 8–14]. This is mainly attributable to differences in study populations and the heterogeneity of its definition. A higher prevalence of MINOCA was found in younger patients (58.8% vs 61.3%, *p* < 0.001), females (43% vs 24%, *p* < 0.001), non-white patients (25% vs 12%, *p* < 0.0001) and in patients presenting with non-STEMI (78% vs 51%, *p* < 0.0001), compared to AMI with obstructive CAD [1, 8, 10, 11, 14, 15].

**Table 2:** Possible underlying aetiologies for myocardial ischaemia with non-obstructive coronary arteries

1. Coronary disorders	Spontaneous coronary artery dissection
	Plaque disruption
	Coronary spasm
	Microvascular dysfunction
	Coronary thrombus/embolus
2. Myocardial disorders	Myocarditis
	Takotsubo cardiomyopathy
	Hypertensive heart disease
	Other cardiomyopathies (e.g. tachycardiomyopathy or use of cardiotoxins/chemotherapeutic agents)
3. Non-cardiac disorders	Stroke
	Pulmonary embolism
	Sepsis
	Adult respiratory distress syndrome
	End-stage renal failure

The VIRGO study [15] also showed that women were 5 times more likely to have MINOCA than men, and that these MINOCA patients had fewer traditional cardiac risk factors, but more often had unconventional risk factors, such as (prior) drug use, hypercoagulability syndrome, venous thromboembolism and autoimmune disorders. Female AMI patients with obstructive CAD were more likely to be menopausal or to have a history of gestational diabetes mellitus compared to those with MINOCA.

Although MINOCA patients have a lower cardiac risk profile, there are conflicting data regarding their prognosis. Safdar et al. described similar functional and psychosocial outcomes. In addition, similar 1‑ and 12-month mortality in both MINOCA and AMI with obstructive CAD [1-month: 1.1% and 1.7% (*p* = 0.43); 12-month: 0.6% and 2.3% (*p* = 0.68), respectively] were found, whereas Pasupathy et al. reported that mortality rates were significantly lower in the MINOCA group compared to AMI with obstructive CAD [in-hospital: 1.1% and 3.2% (*p* = 0.001); 12-month 3.5% and 6.7% (*p* = 0.003), respectively] [1, 15].

An interesting finding by Bainey et al. was that the 1ne-year composite of death and/or reinfarction rate among MINOCA patients with no angiographic evidence of CAD was significantly lower than in MINOCA patients with stenosis <50% (3.9% and 6.1%, [*p* = 0.028], respectively) [16]. In relation to this, independent predictors of adverse outcome were three-vessel disease or left main stem involvement (stenoses ≥30% but <50%), high C‑reactive protein at hospital admission and elevated high-sensitivity cardiac troponin T levels [17, 18].

The previously mentioned data should be interpreted with caution since the outcome of MINOCA strongly depends on the underlying cause. Recently, the prognostic role of cardiac magnetic resonance imaging (CMR) was assessed in MINOCA patients. It was found that a CMR diagnosis of cardiomyopathy was an independent predictor for mortality, whereas a diagnosis of MI, myocarditis or a normal CMR was not [19].

To reveal the exact underlying aetiology of MINOCA, a thorough patient history, physical examination, laboratory testing, imaging and invasive measurements are needed, since MINOCA should be considered as a working diagnosis.

## Cardiac causes of MINOCA: coronary disorders

### Plaque disruption and plaque erosion

The most common pathologies associated with an ACS are plaque rupture, erosion and calcified nodules which are present in 44%, 31% and 8% respectively [20].

Plaque formation starts with the formation of fatty streaks and intimal thickening, leading to fibrous cap atheroma and eventually to fibrous cap thinning. This so-called thin-cap fibroatheroma can rupture.

In plaque erosion, there is an abundance of smooth muscle cells without an extensive necrotic core, haemorrhage or calcification. It differs from plaque rupture, as there is an absence of fibrous cap disruption.

Identification of vulnerable plaques on coronary angiography can be challenging. Computed tomography (CT) angiography and intravascular coronary imaging could play an important role in finding these plaques in the future. Near-infrared spectroscopy-intravascular ultrasound (IVUS) may help to quantify the lipid content of the coronary plaque and could potentially be an important tool to predict future events. Besides, this imaging modality could possibly distinguish whether the MINOCA event is caused by a vulnerable plaque that has ruptured or whether CAD is absent [21]. However, data on intravascular imaging in MINOCA patients is still sparse. In a prospective optical coherence tomography (OCT) study among 38 MINOCA patients, coronary plaque disruption and thrombus were present in 24% and 18%, respectively [22]. Reynolds et al. found similar results with IVUS in women with MINOCA, since plaque disruption was observed in 38% [23].

Treatment of MINOCA caused by plaque disruption or plaque erosion should be managed according to standard treatment recommendations for ACS [3].

### Spontaneous coronary artery dissection

Spontaneous coronary artery dissection (SCAD) is a rare cause of ACS, characterised by a non-traumatic and non-iatrogenic separation of the coronary arterial wall with the creation of a false lumen filled with intramural haematoma [24].

SCAD is associated with younger age (~50 years), female gender (~90%), fibromuscular dysplasia (FMD), pregnancy and the peripartum period in the absence of conventional risk factors for coronary heart disease. The estimated prevalence of SCAD in ACS patients is 1.7–4%. However, in women <50 years of age presenting with ACS, the prevalence could be up to 25% [24, 25]. With increasing awareness of SCAD and the more widespread use of intravascular imaging, the diagnosis SCAD seems to be made more frequently nowadays.

A small proportion of SCAD cases is associated with connective tissue disease such as Marfan or Ehlers-Danlos syndrome [26]. Furthermore, precipitating stressors such as emotional stress, extreme Valsalva-type manoeuvres and the induction of coronary spasm, can provoke the acute SCAD event [24, 27, 28].

SCAD patients usually present with symptoms and signs of ACS. Most cases are diagnosed at the time of coronary angiography with the presence of a radiolucent flap, dual lumen and contrast staining [29].

After the diagnosis of SCAD has been made, conservative management based on expert opinions should be preferred [30–33]. In patients with ongoing ischaemia or haemodynamic instability, coronary revascularisation might be considered. However, this can be challenging due to the fragility of the vessel wall and is associated with high revascularisation failure rates. At follow-up, routine recurrent coronary angiography to determine SCAD healing should be avoided as the benefit does not outweigh the potential risks (e.g. iatrogenic dissections). Further imaging to detect extra-coronary arteriopathies is advised, given the relationship between SCAD and FMD.

There are no guidelines regarding the optimal medical management of SCAD, since randomised controlled trials are lacking. The role of antiplatelet therapy in SCAD remains controversial, since these agents potentially increase the bleeding risk [28]. In contrast, others believe that, since the intimal tear in SCAD can be prothrombotic, dual antiplatelet therapy could be beneficial [24]. Lipid-lowering therapy should only be prescribed to those patients with (pre-existing) dyslipidaemia, since atherosclerosis in SCAD is mostly absent and a small retrospective study demonstrated potentially higher SCAD recurrence with statins [34]. Ongoing prospective studies may further evaluate the usefulness and effects of medical therapy (Clinical Trials NCT02188069 and NCT02008786).

### Coronary artery spasm

Vasospastic angina (VSA) occurs in 28% of patients presenting with MINOCA [1]. However, earlier studies evaluating VSA varied since there was no clear definition of VSA. The Coronary Vasomotion Disorders International Study Group (COVADIS) was established to internationally unify the diagnostic criteria for VSA. These criteria included three core elements, namely (1) nitrate-responsive angina, (2) transient ischaemic electrocardiogram (ECG) changes and (3) angiographic evidence of coronary artery spasm (>90% constriction). In the case of coronary microvascular spasm, no epicardial spasm is present during coronary provocation tests, but ECG changes and recognisable angina symptoms should be present.

Coronary provocation tests with acetylcholine or ergonovine are not routinely performed, since they are thought to be potentially dangerous. However, Montone et al. demonstrated in 80 MINOCA patients that this test could be performed safely directly after coronary angiography. A positive test was found in 37 (46.2%) of the patients and was associated with worse prognosis [35]. Furthermore, a systematic review by Ciliberti et al. in 9444 patients showed that the occurrence of both major (such as ventricular tachycardia or ventricular fibrillation) and minor (such as transient bradycardia, advanced atrioventricular block or paroxysmal atrial fibrillation) complications of pharmacological testing with acetylcholine or ergonovine was low (0.8% and 4.7%, respectively) [36].

Patients with confirmed VSA can be treated with calcium channel blockers and nitrates, with the former shown to be an independent predictor of survival without MI [37].

### Coronary microvascular dysfunction

COVADIS lists the diagnostic criteria for coronary microvascular angina (MVA) due to coronary microvascular dysfunction (MVD) as follows: (1) presence of symptoms suggestive of myocardial ischaemia, (2) objective documentation of myocardial ischaemia, as assessed by currently available techniques, (3) absence of obstructive CAD (stenosis <50%), and (4) confirmation of a reduced coronary blood flow reserve and/or inducible microvascular spasm [38].

The exact prevalence of MVA is unknown, but its incidence in postmenopausal women is high [39]. Several studies describe an occurrence rate of up to 50% in patients with chest pain and non-obstructive coronary arteries [39, 40]. However, there are large differences between studies in relation to the definition of MVD and the use of different diagnostic techniques.

Overall, the prognosis of patients with MVA is comparable to that of patients with obstructive CAD. They continue to have persistent symptoms, have a high prevalence of atherosclerosis, undergo repeat coronary angiographies, and they suffer from greater functional limitations [41, 42]. Besides, higher all-cause mortality rates were observed compared to a reference population without ischaemic heart disease [43].

Coronary microcirculation can be assessed by various invasive and non-invasive techniques (e.g. positron emission tomography, CMR and transthoracic Doppler echocardiography). An impaired coronary flow reserve (CFR), documentation of coronary microvascular spasm, abnormal coronary microvascular resistance (IMR), or a coronary slow flow phenomenon can be objectivised by coronary angiography. A newer technique to assess MVD is the measurement of hyperaemic absolute coronary flow and resistance using thermodilution with a continuous intravenous saline infusion. This technique is operator independent, more reliable and specific compared to CFR and IMR. Recently, it was found to be feasible, safe and reproducible, but its value in MVD is currently unknown [44].

### Coronary thrombus or embolism

In practice, patients presenting with AMI with non-obstructive coronary arteries are stigmatised as having an AMI due to a coronary thrombus or embolus, without a solid explanation. Confirming this diagnosis can be challenging.

Coronary thrombi or emboli may arise from acquired or inherited thromboembolic disorders. Examples of acquired thromboembolic disorders include atrial fibrillation, a left ventricular thrombus, valvular heart disease, malignancy-associated thrombophilia, antiphospholipid syndrome and systemic lupus erythematosus. Hereditary causes of thromboembolism are factor V Leiden, protein C or S deficiency, antithrombin deficiency, or hyperhomocysteinaemia [45, 46]. Thrombophilia screening yields positive results in approximately 14% [1]. Factor V Leiden was the most prevalent inherited thrombotic disorder. However, this is based on small-scale and outdated trials [45–51].

Furthermore, MINOCA might be caused by a paradoxical embolism due to right-left shunting [2]. However, this may be the case in only a very small subset of MINOCA patients and clinical relevance seems limited.

## Cardiac causes: myocardial disorders

### Takotsubo cardiomyopathy

Takotsubo cardiomyopathy is also known as stress cardiomyopathy or ‘broken heart syndrome’; its presentation is similar to that of AMI, but with no obstructive CAD or plaque rupture on angiography. It usually affects postmenopausal female patients and is mostly triggered by an intense emotional or extensive physical trigger. It is characterised by transient, often large, regional left ventricular systolic dysfunction with, in the most common form (81.7%), akinesia of almost the whole heart and hyperkinesia of the basal walls. Furthermore, midventricular, basal and focal forms of Takotsubo cardiomyopathy have been described [52].

The exact underlying pathophysiological mechanism in Takotsubo cardiomyopathy remains unclear. A combination of catecholamine excess, coronary artery spasm and MVD may play a role. It can be difficult to distinguish Takotsubo cardiomyopathy from AMI or acute myocarditis; hence coronary angiography and left ventricular angiography or echocardiography are necessary to confirm the diagnosis. On CMR, myocardial oedema is commonly seen on T2-weighted sequences without detectable myocardial necrosis after late gadolinium enhancement (LGE), which distinguishes it from myocarditis.

Although patients with Takotsubo cardiomyopathy recover spontaneously within several weeks, the in-hospital and long-term adverse outcomes are similar to those of AMI. More interestingly, deformation, fibrosis and metabolic indices remain impaired long after left ventricular ejection fraction is recovered, which results in persistent heart failure symptoms [52, 53].

Currently, there are no guidelines on optimal medical treatment and its duration. Beta-blocker therapy can be useful to achieve adrenergic blockade, and other conventional heart failure therapies might be considered.

### Myocarditis

**Fig. 1:**
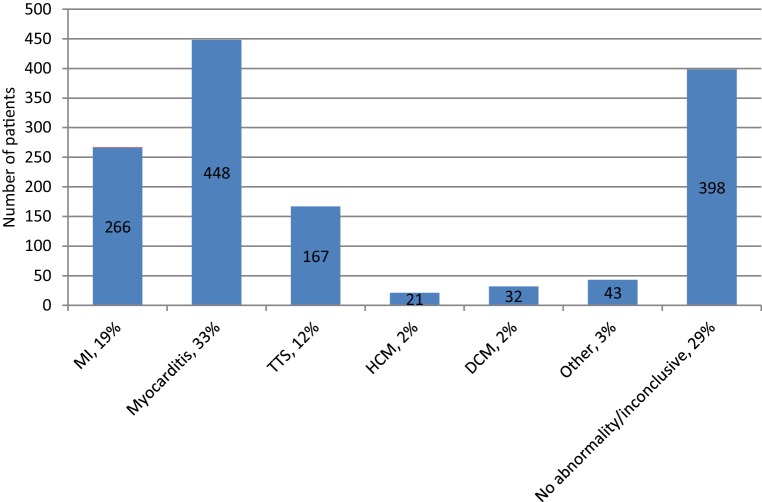
Diagnosis made by cardiac magnetic resonance imaging in patients with myocardial ischaemia with non-obstructive coronary arteries.* MI* myocardial infarction, *TTS* Takotsubo cardiomyopathy, *HCM* hypertrophic cardiomyopathy, *DCM* dilated cardiomyopathy

Myocarditis is an inflammatory disease of the cardiac muscle that is caused by a variety of infectious (e.g. adenoviruses, parvovirus B19, human herpesvirus 6 and Coxsackie virus) and non-infectious conditions (e.g. immune-mediated or toxic). The clinical presentation of acute myocarditis varies widely, ranging from fatigue and chest pain to cardiogenic shock and sudden death. Myocarditis in MINOCA is common, with a mean prevalence of 33% (Fig. 1; [1, 54, 55]). Recognition is important, since myocarditis can deteriorate into fulminant heart failure or even end-stage dilated cardiomyopathy requiring a left ventricular assistant device or heart transplantation. Especially giant cell myocarditis is associated with poor clinical outcomes [56].

CMR can be useful in making the diagnosis of myocarditis, but endomyocardial biopsy should be the gold standard for the diagnosis of definite myocarditis. The timing of these additional investigations is crucial, since myocarditis resolves in approximately 50% of patients within 2–4 weeks [56]. Findings on CMR include patchy, mid-wall, or epicardial oedema on T2-weighted sequences and mid-wall to epicardial scar after LGE and can be clearly distinguished from changes related to AMI (fibrosis).

Conventional treatment of myocarditis in patients with haemodynamically stable heart failure consists of diuretics, angiotensin-converting enzyme inhibitors or angiotensin receptor blockade, and beta-adrenergic blockade [56]. In animal models with myocarditis, non-steroidal anti-inflammatory drugs were found not to be effective. Moreover, they were associated with heart failure exacerbation and increased mortality [57, 58]. Patients with haemodynamically unstable heart failure may require intravenous inotropic agents or mechanical cardiopulmonary support. In the case of biopsy-proven infection-negative myocarditis, immunosuppressive therapy can be considered in specific autoimmune forms.

## Non-cardiac causes

Extra-cardiac causes of MINOCA which can result in myocardial injury include (PE), (end-stage) renal failure, sepsis, stroke and other forms of type 2 MI such as anaemia and hyperthyroidism. They all can be associated with chest pain, elevated cardiac enzymes and ECG changes. If PE is suspected, evaluation with the help of the Wells score, D‑dimer concentration, pulmonary CT angiography or ventilation/perfusion scintigraphy should be performed based on patient-specific presentation.

## Management of MINOCA: proposal of a diagnostic algorithm

The dynamic working diagnosis MINOCA could be made in those patients with suspected AMI, non-obstructive coronary arteries and no clinically overt cause for the acute presentation.

Consequently clinicians should be encouraged to start further evaluation. Stigmatisation of these patients, as having an MI due to coronary thromboembolism or as having non-cardiac chest pain, must be avoided. If the true underlying mechanism for the event has been diagnosed, the working diagnosis MINOCA should be discarded and appropriate treatment should be started and related to the underlying mechanism.

The interventional cardiologist is the first to be confronted with MINOCA at the catheterisation laboratory. Left ventricular angiography or echocardiography should be performed directly after coronary angiography to detect wall motion abnormalities, predominantly to reveal signs of Takotsubo cardiomyopathy.

If, after coronary angiography, the cause of MINOCA is still unknown, re-evaluation by the use of a thorough patient history, physical examination and laboratory assessment should be done, predominantly to exclude non-cardiac causes, various types of type II MI and non-ischaemic mechanisms of the myocyte injury (e.g. myocarditis).

Traditional cardiovascular risk factors for coronary heart disease may imply concealed atherosclerosis and thus endothelial dysfunction, which is a predictive factor for coronary artery spasm. In contrast, SCAD patients have fewer traditional cardiovascular risk factors but this should be strongly considered in young female patients. Elevated inflammatory parameters or elevated D‑dimer levels may suggest myocarditis or PE, respectively. A positive family or personal history for hypercoagulability may lead to suspicion of hereditary or acquired coagulation disturbances.

Non-invasive imaging plays a pivotal role in the detection of the underlying cause for MINOCA. Echocardiography is essential in the work-up of MINOCA to assess any form of structural heart disease, or the presence of ASD, intracardiac thrombus, myocardial tumour or myxoma. Furthermore, CMR plays an important role. Early CMR can differentiate between myocardial inflammation, fibrosis and myocardial function by T1- and T2-weighted imaging, LGE and (ECG-gated) cine imaging. Thirteen studies have evaluated the diagnostic yield of CMR and were able to find a definitive diagnosis in 71% of the patients (19% MI, 33% myocarditis, 12% Takotsubo cardiomyopathy, 2% hypertrophic cardiomyopathy, 2% dilated cardiomyopathy, 3% other), as illustrated in Fig. 1 [4, 19, 54, 59–68]. Based on these observations, we recommend routine examination with CMR within 4 weeks after hospital admission.

**Fig. 2:**
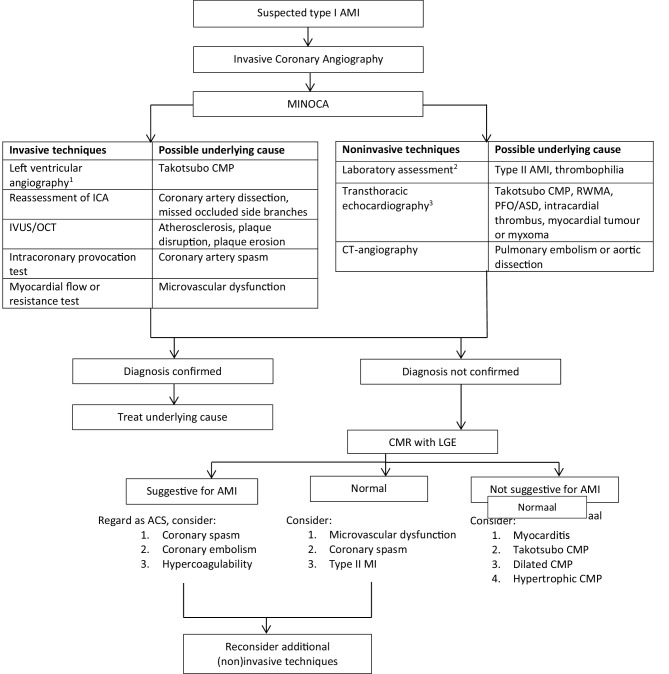
Proposal for a diagnostic algorithm in patients with myocardial ischaemia with non-obstructive coronary arteries. ^a^Unless renal function <35 ml/min per 1.73 m^2^. ^b^Haemoglobin, C‑reactive protein, leucocytes, oxygen saturation, D‑dimers, (NT-pro) brain natriuretic peptide. ^c^ Within 48 h. *AMI* acute myocardial infarction, *MINOCA* myocardial ischaemia with non-obstructive coronary arteries, *ICA* invasive coronary angiography, *CMP* cardiomyopathy, *IVUS* intravascular ultrasound, *OCT* optical coherence tomography, *RWMA* regional wall motion abnormalities, *PFO* patent foramen ovale, *ASD* atrial septal defect, *CT* computed tomography, *CMR* cardiac magnetic resonance imaging, *LGE* late gadolinium enhancement. *ACS* acute coronary syndrome

However, in 8–67% of patients no abnormalities could be found, which leads to a therapeutic dilemma for clinicians [4, 23, 54, 59, 62, 64]. In these patients, additional investigations such as those mentioned in Fig. 2 may be considered.

In select cases, if no cause can be found, it can be useful to perform CT angiography and/or intracoronary reactivity testing during the index procedure or a second procedure in the outpatient clinic to reveal the underlying cause of MINOCA. We recommend this if:

- There remains uncertainty regarding the presence of coronary atherosclerosis or (spontaneous) coronary artery dissection (CT angiography)
- There is a need to detect the degree of coronary atherosclerosis (CT angiography)
- There is a high suspicion of microvascular CAD (intracoronary reactivity testing)
- There is a high suspicion of VSA (intracoronary reactivity testing)

At the discretion of the interventional cardiologist, IVUS or OCT can be performed to determine the presence of atherosclerosis, atherosclerotic plaque disruption, plaque erosion, coronary dissection and coronary thrombosis. In addition, vulnerable plaques can be identified by measuring the fibrous cap thickness and the presence of a large necrotic core. It must be noted, as already mentioned, that coronary plaque disruption and thrombus are highly prevalent in MINOCA [22]. Since the diagnosis of plaque disruption has potential therapeutic implications, the use of intravascular imaging is recommended. The herewith associated higher costs, need for expertise and the extra time needed in the catheterisation laboratory should be taken into consideration.

To evaluate MVA or VSA, a combination of intracoronary interventional diagnostic procedures can be performed directly during the index procedure or a second procedure to assess the CFR (abnormal <2.0), IMR (abnormal ≥25) or FFR (abnormal ≤0.80). If coronary MVD is absent [negative CFR and IMR, in the absence of significant epicardial stenosis (negative FFR)], acetylcholine testing can be performed to reveal epicardial or microvascular spasm [69]. This combined invasive diagnostic approach, including medical therapy, was recently evaluated in the CorMicA trial in patients with stable angina pectoris. It was concluded that this approach improves angina symptoms and quality of life [70].

These techniques are safe in experienced hands. However, one should be aware of the potential complications (e.g. local bleeding complications, coronary artery dissection or perforation, acute kidney injury and stroke). Furthermore, CFR is highly influenced by age, blood pressure, heart frequency and contractility. Besides, it can be difficult to obtain good signals using the Doppler wire.

Altogether, both intravascular imaging and functional testing for detecting vasospasm or microvascular resistance play an important role in detecting the true mechanism of MINOCA. What kind of testing is most appropriate depends on the clinical presentation and the hospital resources. For example, intracoronary provocation testing may be the first choice in a patient with nocturnal angina pectoris and transient ST-elevation presenting with non-obstructive coronary arteries; on the other hand, if a patient with multiple traditional cardiac risk factors is diagnosed with subcritical stenoses, intracoronary imaging, and CFR and IMR measurements provide the most information (e.g. degree of atherosclerosis, plaque rupture or vulnerable plaque).

## Future perspectives

The Stockholm Myocardial Infarction with Normal Coronaries (SMINC-2) study will provide insight into whether or not early CMR can make a reliable diagnosis in more than 70% of all MINOCA patients (Clinical Trials NCT02318498) [71]. Furthermore, the ongoing MINOCA BAT (Clinical Trials NCT03686696) aims to provide information on the usefulness of beta blockers and angiotensin-converting enzyme inhibitors or angiotensin receptor blockers in 3500 MINOCA patients.

## Limitations

Several limitations should be addressed. First, the term MINOCA can be interpreted in several ways, and thus studies vary widely regarding the inclusion criteria of MINOCA. Many terms have been coined to describe patient with non-obstructive coronary arteries during an ACS. As the Dutch ACS working group we think use of an additional term will only lead to even less clarity, and thereby suggest using the term MINOCA as a dynamic working diagnosis to describe all possible underlying causes (i.e. coronary, myocardial and non-coronary disorders).

Secondly, the 50% angiographic stenosis threshold in MINOCA is somewhat arbitrary, since it was shown that the FFR was positive in a quarter of the patients with angiographically considered non-obstructive coronary arteries [72]. Although data on FFR testing in MINOCA patients is sparse, we agree with the American Heart Association that, if FFR is used, only patients with FFR findings >0.80 should be included in a working diagnosis of MINOCA [73]. Besides, for prognostic purposes it would be important to subdivide the MINOCA patients according to their angiographic coronary status into those with normal coronary arteries and mild CAD in future studies.

Third, since the MINOCA population is per definition heterogeneous, it is a challenge to make a clear-cut diagnostic pathway for every MINOCA case. For this reason, we chose to make a general algorithm including all potentially useful diagnostic modalities. Depending on the clinical presentation and the hospital resources, a patient-specific diagnostic approach should be made. However, CMR should play an important role in this approach given the high diagnostic yield.

## Conclusion

MINOCA is a common clinical entity in patients presenting with AMI and represents many possible aetiologies that can be challenging to detect. By proposing a clinical diagnostic algorithm, we aim to encourage the clinician to find the underlying cause of MINOCA, since MINOCA should be regarded as a dynamic working diagnosis including coronary, myocardial and non-coronary disorders.
