# Correction to: Guidelines for the management of myocardial infarction/injury with non-obstructive coronary arteries (MINOCA): a position paper from the Dutch ACS working group

**DOI:** 10.1007/s12471-019-01358-0

**Published:** 2019-12-11

**Authors:** T. F. S. Pustjens, Y. Appelman, P. Damman, J. M. ten Berg, J. W. Jukema, R. J. de Winter, W. R. P. Agema, M. L. J. van der Wielen, F. Arslan, S. Rasoul, A. W. J. van ’t Hof

**Affiliations:** 1 Department of Cardiology, Zuyderland Medical Centre, Heerlen, The Netherlands; 2 Department of Cardiology, location VU University Medical Centre, Amsterdam UMC, Amsterdam, The Netherlands; 3 Department of Cardiology, Radboud University Medical Centre, Nijmegen, The Netherlands; 4 Department of Cardiology, St Antonius Hospital, Nieuwegein, The Netherlands; 5 Department of Cardiology, Leiden University Medical Centre, Leiden, The Netherlands; 6 Department of Cardiology, location Academic Medical Centre, Amsterdam UMC, Amsterdam, The Netherlands; 7 Department of Cardiology, Jeroen Bosch Hospital, ’s-Hertogenbosch, The Netherlands; 8 Department of Cardiology, location Bethesda, Treant Zorggroep, Hoogeveen, The Netherlands; 9 Department of Cardiology, Maastricht University Medical Centre, Maastricht, The Netherlands; 10 Department of Cardiology, Isala Hospital, Zwolle, The Netherlands

**Correction to:**

**Neth Heart J 2019**

10.1007/s12471-019-01344-6

The reference to the term acute coronary syndrome with normal or near-normal (non-obstructive) coronary arteries (ACSNNOCA) from Manolis et al. (2018) was inadvertently omitted to the original published article. Therefore, the following reference should be added to our paper:

- Manolis AS, Manolis AA, Manolis TA, Melita H. Acute coronary syndromes in patients with angiographically normal or near normal (non-obstructive) coronary arteries. Trends Cardiovasc Med. 2018;28(8):541–51. 10.1016/j.tcm.2018.05.006.

The authors would like to apologise for any inconvenience caused.

